# Linked Clusters of SARS-CoV-2 Variant B.1.351 — Maryland, January–February 2021

**DOI:** 10.15585/mmwr.mm7017a5

**Published:** 2021-04-30

**Authors:** Kenneth A. Feder, Marcia Pearlowitz, Alexandra Goode, Monique Duwell, Thelonious W. Williams, Ping An Chen-Carrington, Ami Patel, Catherine Dominguez, Eric N. Keller, Liore Klein, Alessandra Rivera-Colon, Heba H. Mostafa, C. Paul Morris, Neil Patel, Anna M. Schauer, Robert Myers, David Blythe, Katherine A. Feldman

**Affiliations:** ^1^Maryland Department of Health; ^2^Epidemic Intelligence Service, CDC; ^3^CDC Foundation, Atlanta, Georgia; ^4^Department of Pathology, Division of Medical Microbiology, Johns Hopkins University, Baltimore, Maryland; ^5^National Institute of Allergy and Infectious Diseases, National Institutes of Health, Bethesda, Maryland; ^6^Baltimore County Department of Health, Maryland; ^7^Baltimore City Department of Health, Maryland.

In late January 2021, a clinical laboratory notified the Maryland Department of Health (MDH) that the SARS-CoV-2 variant of concern B.1.351 had been identified in a specimen collected from a Maryland resident with COVID-19 (*1*). The SARS-CoV-2 B.1.351 lineage was first identified in South Africa (*2*) and might be neutralized less effectively by antibodies produced after vaccination or natural infection with other strains (*3*–*6*). To limit SARS-CoV-2 chains of transmission associated with this index patient, MDH used contact tracing to identify the source of infection and any linked infections among other persons. The investigation identified two linked clusters of SARS-CoV-2 infection that included 17 patients. Three additional specimens from these clusters were sequenced; all three had the B.1.351 variant and all sequences were closely related to the sequence from the index patient’s specimen. Among the 17 patients identified, none reported recent international travel or contact with international travelers. Two patients, including the index patient, had received the first of a 2-dose COVID-19 vaccination series in the 2 weeks before their likely exposure; one additional patient had a confirmed SARS-CoV-2 infection 5 months before exposure. Two patients were hospitalized with COVID-19, and one died. These first identified linked clusters of B.1.351 infections in the United States with no apparent link to international travel highlight the importance of expanding the scope and volume of genetic surveillance programs to identify variants, completing contact investigations for SARS-CoV-2 infections, and using universal prevention strategies, including vaccination, masking, and physical distancing, to control the spread of variants of concern.

Case investigation, contact elicitation (following CDC guidelines for defining close contacts) (*7*,*8*), and contact tracing were conducted for the index patient immediately after the initial diagnostic test result before the sequencing results were available; in Maryland, this is standard procedure for all persons with COVID-19 diagnosed by a SARS-CoV-2 antigen test or nucleic acid amplification test (NAAT) (including reverse transcription–polymerase chain reaction [RT-PCR]). This process was conducted for all COVID-19 cases identified from among the index patient’s contacts until no additional cases in the transmission chain could be identified. Interviews of persons with positive test results and their contacts were documented in a central data management system via a scripted electronic form and as audio recordings. For this investigation, electronic forms and recordings were reviewed, and available specimens from associated patients were sequenced by the Maryland Public Health Laboratory. This activity was reviewed by CDC and con­ducted consistent with applicable federal law and policy.*

The index patient reported two potential exposure settings that might have led to SARS-CoV-2 infection, including a workplace (3 days before symptom onset) and an indoor social gathering (2 days before symptom onset). The patient’s workplace was excluded as the source of infection: investigation of the workplace identified no close contacts or high-risk exposures and no additional employees with SARS-CoV-2 infections. The index patient also attended an indoor social gathering with six other persons 2 days before symptom onset; the event lasted several hours, and attendees removed masks while eating. Review of contact tracing records for the six other attendees found that all six received positive SARS-CoV-2 antigen or NAAT test results, with specimen collection dates ranging from 3 to 13 days after the gathering. Five of the six attendees had symptomatic COVID-19; symptom onsets ranged from the day of the gathering through 8 days afterward (Figure 1). One attendee named two additional close contacts, both of whom received negative NAAT test results. The index patient identified one additional close contact during the infectious period. The final close contact named by the index patient never experienced symptoms of COVID-19 and received a negative SARS-CoV-2 NAAT test result as well as a negative SARS-CoV-2 immunoglobulin G antibody test result.

**FIGURE 1:**
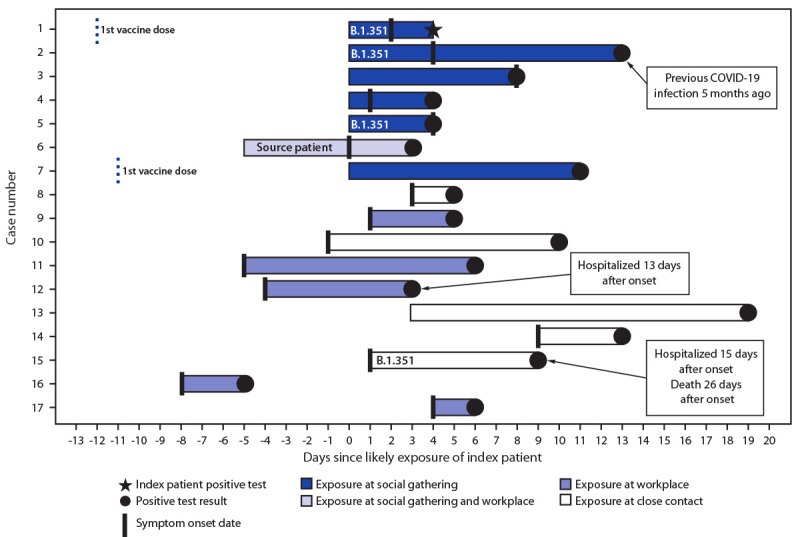
Timelines of exposures, symptom onsets, and SARS-CoV-2–positive test results,* including characteristics of cases associated with a B.1.351 variant investigation**^†^** — Maryland, January–February 2021 * Bars represent the number of days from either the individual patient’s exposure date or symptom onset date to the date of positive test result. Exposure dates and onset dates are missing for some patients. Among patients with both exposure and onset dates available, the earlier date is used in this calculation. ^†^ The index patient represented the first case identified during this investigation. The source patient had the earliest self-reported onset date among attendees of the social gathering and was identified as a possible source of infection at the gathering.

Among attendees at the social gathering reported by the index patient, the earliest self-reported illness onset was on the date of the social gathering. That person was identified as a possible source of infection for the other persons who attended the gathering. Retrospective review of this source patient’s interview revealed that the patient’s workplace was a business that had been reported through an anonymous tip line established for reporting COVID-19 safety concerns; several employees working while displaying symptoms consistent with COVID-19 were reported. The local health department initiated an outbreak investigation at the source patient’s workplace, which found that employees worked in close quarters where physical distancing was not possible and that some employees had attended work while experiencing COVID-19–like symptoms. This workplace had seven employees (including the source patient), six of whom were symptomatic and received positive SARS-CoV-2 antigen or NAAT test results. Symptom onset dates occurred over a period of 12 days; symptom onset in three patients preceded that of the source patient, and two occurred later. The seventh employee never experienced symptoms and received two negative test results during this period. The six employees who received positive SARS-CoV-2 test results, including the source patient, named eight nonwork close contacts (in addition to those already identified from the indoor social gathering), five of whom received positive SARS-CoV-2 RT-PCR test results. The three other close contacts never experienced symptoms; NAAT test results were negative for one contact and inconclusive for another, and the third contact was not tested. 

These two linked clusters resulted in a total of 17 laboratory-confirmed SARS-CoV-2 infections, including all seven attendees of the social gathering; six of seven employees of the source patient’s workplace (with the source patient counted in both clusters); and five of 11 close contacts of persons with laboratory-confirmed infection in either setting (Figure 2). No patient reported a history of international travel or close contact with anyone with a history of recent international travel.

**FIGURE 2:**
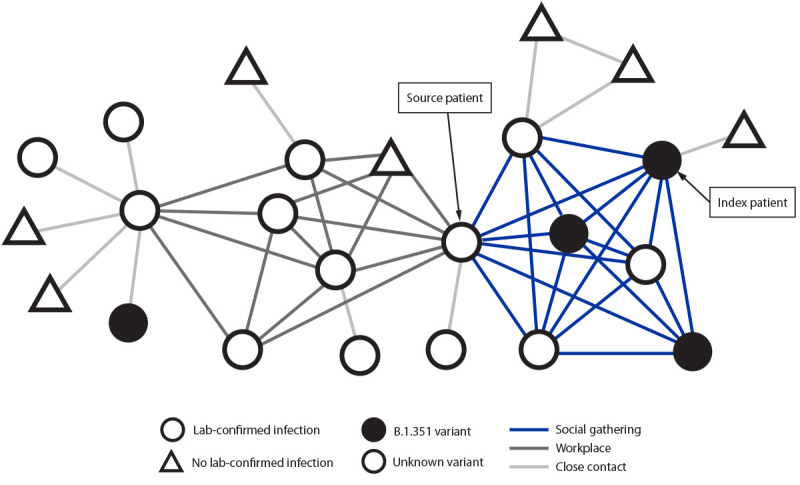
Persons with laboratory-confirmed SARS-CoV-2 infection and asymptomatic contacts without positive SARS-CoV-2 tests* associated with an investigation of B.1.351 variant SARS-CoV-2 infection,† by link type^§^ (N = 24) — Maryland, January–February 2021 * No laboratory-confirmed infection indicates persons named in the case interview who were within 6 ft of the patient for a total of ≥15 minutes over a 24-hour period starting from 2 days before illness or test specimen collection and who did not receive a positive SARS-CoV-2 test result (n = 7). ^†^ Four specimens were sequenced and confirmed to be the B.1.351 variant, including that from the index patient; other specimens were not available for sequencing. ^§^ Before symptom onset, the index patient and six persons attended a social gathering; one of those persons was also connected to a workplace along with six other persons. Close contacts are persons for whom a household or other close connection with a patient was determined. The source patient had the earliest self-reported onset date for an attendee of the social gathering and was identified as a possible source of infection at the gathering.

Four total specimens from these clusters were sequenced, and all were of the B.1.351 lineage, including the index patient’s specimen, two specimens from patients also exclusively associated with the social gathering cluster, and one specimen from a patient exclusively associated with the source patient’s workplace cluster (Figure 3). Two sequences were identical to that of the index specimen. One differed from the index specimen by a single nucleotide polymorphism.

**FIGURE 3:**
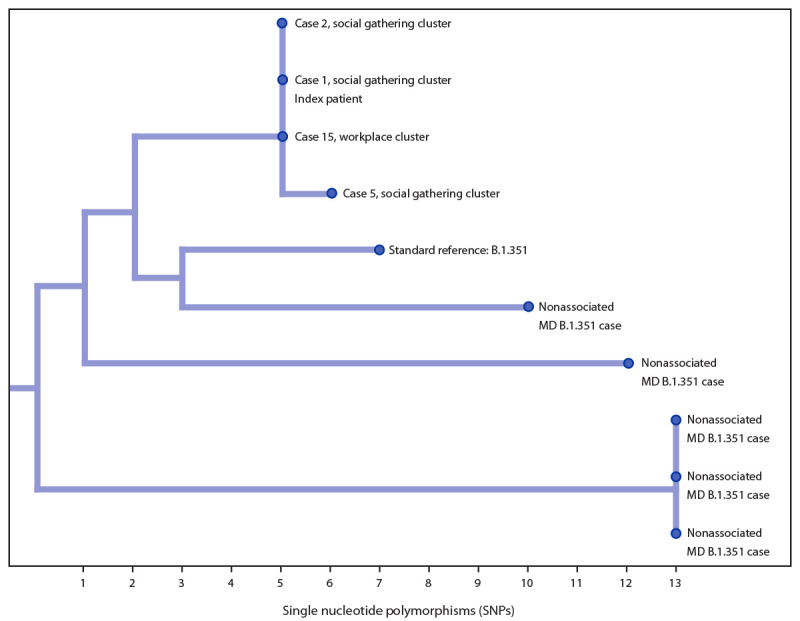
Phylogenetic tree of four investigation-associated B.1.351 lineage specimens* and five other non–investigation-associated B.1.351 specimens sequenced from Maryland resident patients — Maryland, January–February 2021 * Patient case numbers correspond to four of the 17 cases identified in the investigation. X-axis denotes the SNP distance of specimens from the nearest common ancestor of all sequenced Maryland B.1.351 specimens at the time of analysis.

Two patients (aged 42 and 74 years) were hospitalized, including one employee of the source patient’s workplace and one close contact of an employee in that workplace; one of these patients (aged 74 years) died. Neither had a history of vaccination or previous infection. Two symptomatic infections occurred in persons who had received the first of a 2-dose COVID-19 vaccination series 11 and 12 days before exposure, and one symptomatic infection occurred in a person with NAAT-confirmed symptomatic SARS-CoV-2 infection diagnosed approximately 5 months before symptom onset (Figure 1).

## Discussion

This report documents the first identified linked clusters of B.1.351 infections in the United States with no identified link to international travel. Given the rapid spread of B.1.351 and the possible reduced susceptibility to neutralizing antibodies produced after vaccination or infection with other strains (*3*–*6*), this investigation highlights several important points for public health agencies responding to the B.1.351 SARS-CoV-2 variant and other variants of concern.

First, most of the case investigations took place before persons in case clusters were identified as having been infected by the B.1.351 SARS-CoV-2 variant. Genetic sequencing of SARS-CoV-2 specimens usually takes several days beyond the time needed for NAAT testing. Consequently, most successful variant case investigations and contact tracing are conducted before the variant case is identified by sequencing (*9*). Therefore, consistent implementation of best practices for case investigation and contact tracing as well as universal application of prevention strategies, including consistent and correct use of masks, physical distancing, and hand hygiene, are critical to controlling the spread of all SARS-CoV-2 variants, including B.1.351 (*9*).

Second, the index infection was identified in a person whose specimen was sequenced even though no history of international travel was reported. To maximize identification of variants of concern, prioritization of cases with factors that could indicate infection with a variant of concern (e.g., possible reinfection, vaccine failure, travel, and unusual clinical presentations) is important, as is random sequencing of specimens with low NAAT cycle threshold values, which might be more likely to produce a viable sequence; this is the approach currently used by Maryland’s Public Health Laboratory.

Third, practices used by MDH and its local health department counterparts could be particularly useful for other health departments investigating clusters of SARS-CoV-2. These practices include audio recording interviews of persons with cases and their contacts for preservation of information and reinvestigation if needed; searching for the potential source of infection for confirmed cases, in addition to eliciting their exposed contacts to contain the spread (*6*); and establishing an anonymous tip line for COVID-19 safety concerns.

The findings in this report are subject to at least two limitations. First, because not all patients had specimens available for sequencing, some infections could have been associated with a separate SARS-CoV-2 introduction. Second, disclosure of close contacts might have been unreliable, and additional instances of transmission might have been missed.

This investigation identified multiple instances of transmission of the B.1.351 SARS-CoV-2 lineage in Maryland with no identified link to international travel. These findings have implications for public health agencies responding to SARS-CoV-2 variants of concern. Programs might improve detection and tracking of variant cases by expanding the scope and volume of genetic surveillance programs’ sequencing. More generally, the findings highlight the importance of completing contact investigations for SARS-CoV-2 infections and using universal prevention strategies, including vaccination, masking, and physical distancing, to control the spread of variants of concern.

**Summary**

**What is already known about this topic?**

In January 2021, a SARS-CoV-2 specimen from a Maryland resident was determined to be the B.1.351 variant, first identified in South Africa. The SARS-CoV-2 B.1.351 variant might elicit a reduced neutralizing antibody response.

**What is added by this report?**

Investigation identified two linked clusters of SARS-CoV-2 infection, comprising 17 total patients (two were hospitalized and one died) who did not report recent travel. Four patients’ specimens were sequenced; all were the B.1.351 variant.

**What are the implications for public health practice?**

These were the first identified clusters of B.1.351 in the United States with no link to travel. Completed contact investigations, expanded genetic sequencing, and universal prevention strategies, including vaccination, masking, and distance, might prevent the spread of SARS-CoV-2 variants of concern, including B.1.351.

## Footnotes

* 45 C.F.R. part 46.102(l)(2), 21 C.F.R. part 56; 42 U.S.C. Sect. 241(d); 5 U.S.C. Sect. 552a; 44 U.S.C. Sect. 3501 et seq.
